# LONGRANGE® (eprinomectin 5% w/v extended-release injection) efficacy against *Hypoderma lineatum* in an endemic area in southern Italy

**DOI:** 10.1186/s13071-019-3475-y

**Published:** 2019-05-14

**Authors:** Riccardo Paolo Lia, Steffen Rehbein, Alessio Giannelli, Becky Fankhauser, Domenico Otranto

**Affiliations:** 1 Department of Veterinary Medicine, University of Bari, Str. prov. per Casamassima Km 3, Valenzano, 70010 Bari, Italy; 2 Boehringer Ingelheim Vetmedica GmbH, Kathrinenhof Research Center, Walchenseestr. 8–12, 83101 Rohrdorf, Germany; 3 Boehringer Ingelheim Animal Health USA, Inc, 3239 Satellite Blvd, Duluth, GA 30096–4640 USA

**Keywords:** Eprinomectin, Extended-release injection, Efficacy, Warble flies, *Hypoderma lineatum*, Cattle

## Abstract

**Background:**

Despite intensive control measures including governmental campaigns using highly-efficacious systemic insecticides, there is evidence for persisting or recurring bovine *Hypoderma* species populations in parts of Europe, the USA and Canada. The present study evaluated the efficacy of LONGRANGE® (eprinomectin 5% w/v extended-release injection) against the infestation of cattle with *Hypoderma lineatum*, which is considered to be the predominant bovine warble fly in southern Europe and in North America.

**Methods:**

Thirty-six local breed cattle sourced in an endemic area in southern Italy and confirmed positive for *Hypoderma* exposure by ELISA were randomly assigned to three groups of 12 animals each. Cattle of one group served as control and received saline injectable solution, whereas those in the two other groups received LONGRANGE® by subcutaneous injection. LONGRANGE® was administered once, either when *Hypoderma* larvae were expected to be first-instars (L1) or after warbles development, with *Hypoderma* larvae moulting to the second-(L2) and third-(L3) instars. Cattle were checked at intervals for warbles and *Hypoderma* larvae were collected, examined for their viability and morphologically identified. The detection of *Hypoderma* on cattle was terminated when warbles were no longer emerging.

**Results:**

All intact larvae collected were identified as *H. lineatum*. No live larvae were collected from any animal treated with LONGRANGE® while live specimens were sampled from nine of the 12 control cattle (1 to 9 larvae per animal) (*P* = 0.0001 at α = 0.05). LONGRANGE® treatment was well accepted and no adverse events related to treatment or other health problems were observed.

**Conclusions:**

This study confirmed the continued ‘preventive’ (efficacy against migrating L1) and ‘therapeutic’ (efficacy against L2 and L3 in warbles) efficacy of LONGRANGE® against *H. lineatum* infestation of cattle under contemporary field conditions.

## Background

Among the insects parasitizing cattle, warble flies of the genus *Hypoderma* received significant attention when they were common and widespread in the past because of production losses caused by the larvae which develop in large subcutaneous lumps, or so-called warbles, in the backs of cattle. The economic importance in infested cattle results mainly from losses in hide value due to holes left by emerging grubs or scars remaining for several months after emergence, as well as losses in carcass value due to the larval damage to the underlying flesh requiring carcass trimming [1, 2]. The development in the late 1950s of systemic organophosphate insecticides for use in cattle followed by the introduction of the macrocyclic lactones in the 1980s [3, 4] resulted in a marked decrease in the prevalence of bovine hypodermosis in most developed countries in the northern hemisphere and even eradication in some areas following the implementation of area-wide campaigns or national control programs [5, 6]. However, as summarized recently [7], residual populations of bovine *Hypoderma* spp. are still present in Canada and the USA and in several countries of continental Europe.

Three species in the genus *Hypoderma* are parasites of cattle: *H. bovis*, *H. lineatum* and *H. sinense* [8]. The species established in cattle in North America and Europe are *H. bovis*, also referred to as the larger warble fly or northern cattle grub, and *H. lineatum*, the lesser warble fly or common cattle grub. Although both species are still known to occur in North America and Europe [7], only scarce data are available on the current relative distribution of the two cattle warble fly species. This general lack of knowledge about the current prevalence of the two *Hypoderma* species in endemic countries is due to the overall significant decrease in incidence and the preferred use of ELISAs in surveillance programs in place of clinical examination of cattle or of carcasses at abattoirs. Based on the available information, *H. bovis* appears to be more dominant in central, eastern and northern Europe. *Hypoderma lineatum*, however, seems to be the predominant species in North America and southern Europe [9, 10] where it has been rarely reported to infest humans causing creeping subdermal myiasis [11–13] as the host specificity is not absolute.

The life history of both *Hypoderma* species is very similar and although there are some differences in the biology of *H. bovis* and *H. lineatum* (e.g. larval migratory route, winter resting sites in the body, timing of normal annual activity of the adult flies and of normal appearance of warbles in the backs of cattle), the *Hypoderma* species have two important aspects of their life-cycle in common: there is only one generation per year and the entire *Hypoderma* population is resident within the host during the winter [14, 15]. These characteristics are the prerequisite for the efficient control of hypodermosis using systemically acting treatments which kill the *Hypoderma* larvae while migrating through the animal’s body and thus prevent the formation of warbles.

Eprinomectin is the most recently commercialized member of the macrocyclic lactones in large animals. It was developed in the 1990s as a topical treatment for cattle of all classes against nematode endoparasite infections and arthropod ectoparasite infestations including hypodermosis [16–18]. A 5% w/v extended-release injection (LONGRANGE®) of eprinomectin was later developed, mainly for grazing animals [19]. The therapeutic anthelmintic activity of LONGRANGE® is similar to the topical treatment [20–22] but it provides as a major strength a significantly prolonged prevention of infection efficacy against a broad spectrum of gastrointestinal nematodes and lungworms for up to 150 days [23, 24]. Although the efficacy in cattle of LONGRANGE® against *Hypoderma* spp. (including one study with *H. lineatum* infested cattle) has previously been demonstrated [25], the present study was conducted to re-confirm the efficacy of the treatment against contemporary *H. lineatum* given that this species currently appears to be the predominant *Hypoderma* species in cattle in North America and southern Europe.

## Results

No warbles were detected at the first examination of the cattle on January 23rd, 2017 (Day 41); however, one to three warbles were detected initially in three animals on February 2nd, 2017 (Day 55). While no warbles were detected over the course of the study in any of the 12 cattle which were administered LONGRANGE® when the *Hypoderma* larvae were expected to be L1 in December 2016, the number of warble-positive animals in the two other groups increased over time. Warbles were observed in 6 (range, 2–14 warbles) and 9 (range, 1–19 warbles) animals in Group 1, and in 9 (range, 1–18 warbles) and 9 (range, 1–17 warbles) animals in Group 3 on two consecutive occasions (Days 97 and 104, March 20 and 27, 2017). LONGRANGE® was administered accordingly to the Group 3 animals at Day 109. Peak warble counts in both controls (Group 1) and animals which were administered LONGRANGE® at Day 109 (Group 3) were recorded at Day 111 (April 03, 2017) when warbles were recorded in 11 or in 10 animals, respectively, with individual counts ranging in these animals from 1–18 or 1–17, respectively.

**Fig. 1:**
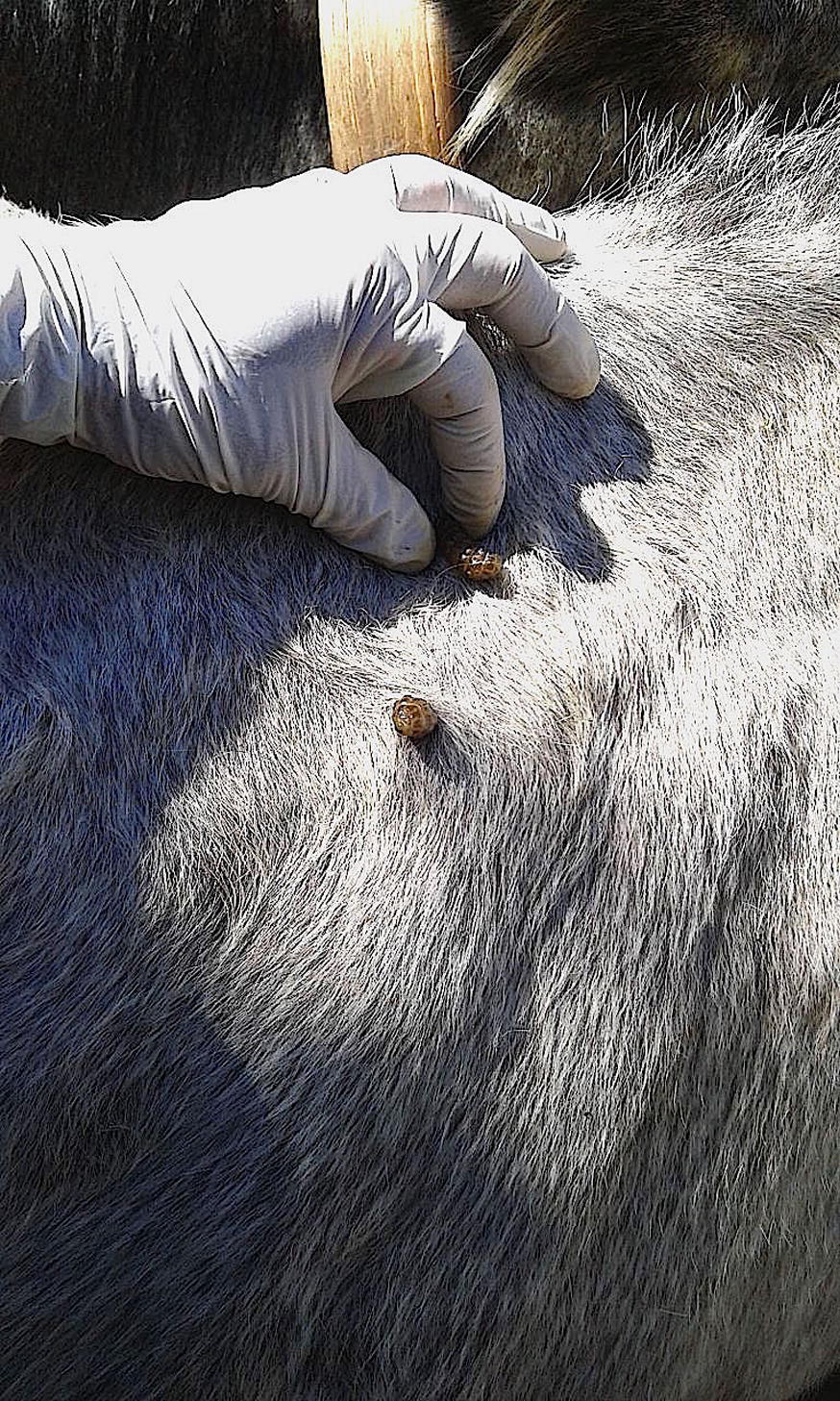
Live *Hypoderma lineatum* third-stage larvae about to emerge manually expressed from the warbles of an untreated control animal

**Fig. 2:**
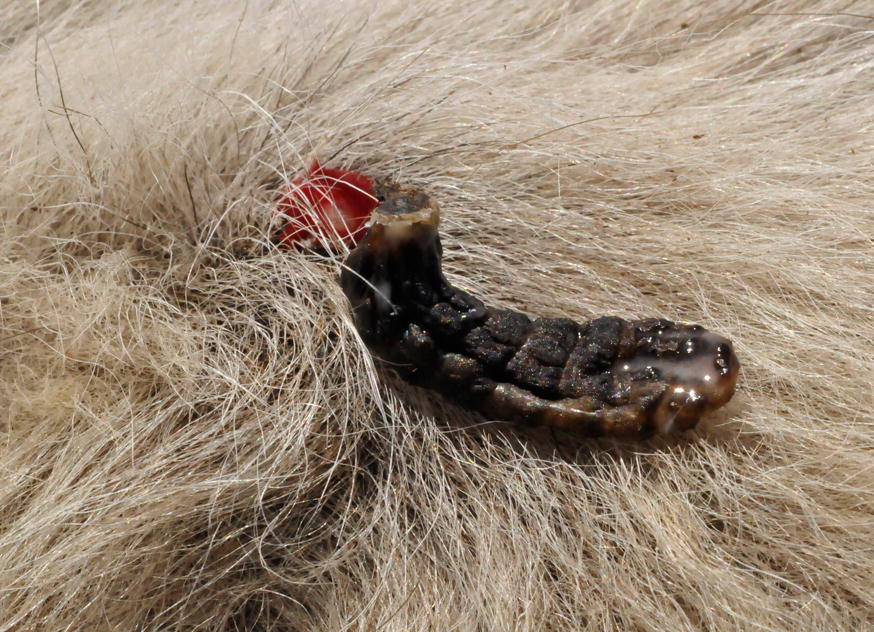
Dead *Hypoderma lineatum* third-instar larva manually removed from a warble of an animal treated with LONGRANGE® at Day 109

By examination of the cattle on a weekly basis from Day 69 to Day 148, a total 35 live *H. lineatum* larvae (4 L2, 31 L3) (Fig. 1) were collected from 9 of the 12 saline-treated control animals (range, 1–9 larvae). No live *Hypoderma* larvae were collected from any of the cattle treated with LONGRANGE® on Day 0 or Day 109; however, a total of 36 dead *Hypoderma* larvae (34 *H. lineatum* L3; 2 *Hypoderma* spp. larvae, unidentifiable to species) (Fig. 2) were removed from seven cattle of the latter group by the conclusion of the study. Cattle in the two LONGRANGE® groups had significantly fewer live larval *H. lineatum* counts (*P* = 0.0001) than the saline control group cattle at α = 0.05 and the percentage efficacy was 100% (Table 1).

**Table 1:** Number of live *Hypoderma lineatum* larvae collected and efficacy of LONGRANGE® against infestation with first-stage, and second- and third-stage larvae of *H. lineatum* of cattle

Treatment group^a^	Count of live *H. lineatum* L2/L3 collected from the study animals		% efficacy^b^	*F-*value*, P*-value^c^
	NI/NG^d^	GM^e^ (range)		
Saline (control)	9/12	2.0 (0–9)	–	–
LONGRANGE® L1	0/12	0	100	*F*_(1,21)_ = 21.94, *P* = 0.0001
LONGRANGE® L2/L3	0/12	0	100	*F*_(1,21)_ = 21.94, *P* = 0.0001

^a^Saline, administered at 1 ml per 50 kg body weight (BWT) once on Day 0 and once on Day 109; LONGRANGE® L1, LONGRANGE® (eprinomectin 5%w/v extended-release injection) administered at 1 ml per 50 kg BWT once on Day 0 during the first larval stage (L1) of development; LONGRANGE® L2/L3, LONGRANGE® administered at 1 ml per 50 kg BWT once on Day 109 during the second and third larval stages (L2/L3) of development

^b^Percentage efficacy = [(C-T)/C] × 100, where T and C are geometric means of the back-transformed least squares means from the statistical model of the LONGRANGE® L1 group or LONGRANGE® L2/L3 group counts (T), and the saline group count (C), respectively

^c^*F-*value, *P*-value = two-sided probability value from analysis of variance on log-counts of LONGRANGE® and saline groups

^d^Number of cattle from which live *H. lineatum* L2/L3 were collected (number infested, NI)/number of cattle in group (NG)

^e^GM = geometric mean

All animals accepted the treatments well as they were reported as normal during the observations 2 and 4 h post-treatment on Day 0 and on Day 109. No adverse experience related to treatment or any other health problems were observed throughout the study.

## Discussion

Results of this study confirm the excellent level of efficacy of eprinomectin when administered as an extended-release injection to cattle naturally infested with the parasitic stages of the warble fly, *H. lineatum*. LONGRANGE® was shown to be efficacious both as a ʻprophylacticʼ treatment for *H. lineatum* larval infestation, i.e. when there is no external evidence of their presence and before causing a decrease in the value of the skin and carcass, and as a ʻtherapeuticʼ treatment, e.g. when warbles have already developed.

According to previous studies [7, 25], serodiagnosis was used to select study animals as it is the only way to detect infested animals before warbles appear in the backs of the animals. Development of warbles in this study was ultimately recorded in 87.5% of the combined controls and animals treated after warbles have already developed. The 87.5% infestation rate is consistent with observations from earlier studies in which 75 to 86.4% of cattle that were positive for anti-Hypoderma antibodies ultimately developed warbles in their backs and is thus in-line with the predictive value of the available ELISAs [7, 25].

A study with a similar design as that reported here was conducted in the same area during the 2006/2007 *Hypoderma* season [7]. In both studies, only anti-*Hypoderma* antibody positive animals were enrolled and were less than one year of age and of local breeds. The overall infestation pattern, in terms of the development of warbles in the backs of the cattle, was similar in the two studies and overlapped with the general seasonality of *H. lineatum* infestation in the Mediterranean region of Europe [26]. However, the first warbles in the present study were detected about one month later than in the previous study (early February *vs* early January), and peak warble counts occurred in early April in the present study while in the previous study they were observed in late February. Such variation within the same geographical area can be related to variation in environmental conditions [26, 27]. The combined control cattle and cattle treated when *H. lineatum* larvae were L2/L3 which were enrolled in the previous (*n* = 22 cattle) and in the present (*n* = 24 cattle) studies had similar anti-*Hypoderma* antibody titres (mean S/P% 264.3 and 287.6, respectively) and a similar percentage of cattle developed warbles in their backs, 86.4 and 87.5%, respectively. However, the cattle enrolled in the 2006/2007 study demonstrated substantially higher warble counts than the cattle enrolled in the present study with peak mean warble counts of 15.2 and 4.8 and individual counts of up to 46 and 18 warbles, respectively, which confirms that titer level and intensity of infestation do not correlate as reported previously [28–30]. The reason for this difference in the warble counts of the animals grazing in the same area is difficult to explain but it may be related to an increase in the general use of macrocyclic lactone products in the cattle population in the area.

## Conclusions

This study confirms the continued excellent efficacy against *H. lineatum* infestation of cattle under contemporary field conditions of LONGRANGE® which has previously been demonstrated to prevent the establishment of nematode infections in cattle for up to 150 days [24]. Therefore, based on the results of this study it can be assumed that LONGRANGE® treatment of cattle for the control of nematode infections at the beginning of the pasture season would also protect animals from hypodermosis when adult warble flies are active and ovipositioning [15, 26, 31].

## Methods

The design of the study was in accordance with and consistent to the World Association for the Advancement of Veterinary Parasitology guideline for evaluating the efficacy of ectoparasiticides against myiasis causing parasites [32]. The study was conducted in compliance with VICH GL9, entitled Good Clinical Practice.

The study was performed as blinded study, i.e. all personnel involved in collecting efficacy data were masked as to the treatment assignment of the animals.

In November 2016, 36 local beef breed cattle (18 Podolica, 1 Romagnola, 17 crossbreed) were sourced from Abriola (Potenza province, Basilicata region, Italy; 40°50′N, 15°81′E; approximately 957 m above sea level). The area is historically known to be endemic for *H. lineatum* [7, 33], and the animals’ exposure to *Hypoderma* spp. was confirmed prior to enrolment in the study by ELISA. Using the IDEXX Bovine Hypodermosis Antibody Test (IDEXX Montpellier SAS, Montpellier, France) all animals tested positive (sample/positive ratio, S/P% ≥ 55) for anti-*Hypoderma* antibodies: S/P% ≥ 55 to < 100, 1 animal; S/P% 100 to < 200, 2 animals; S/P% 200 to < 300, 12 animals; S/P% 300 to < 400, 18 animals; S/P% 400 to < 500, 3 animals). The cattle, 21 male and 15 female, approximately five to eight months of age and weighing initially between 117 and 290 kg, were healthy as confirmed by physical examination at enrolment and had not been previously treated with a macrocyclic lactone product.

The cattle were randomly divided in three groups (i.e. 12 animals for each group) using a unique randomization table generated by the PLAN procedure in SAS v.9.4 based on presentation of animals at Day 0 weighing, and each animal was randomly allocated to one of two indoor loose-pens at the study site. Cattle allocated to Group 1 (control) received saline (sodium chloride 0.9% w/v) injectable solution at 1 ml/50 kg body weight, once on Day 0 (December 13, 2016) and once on Day 109 (April 01, 2017); cattle allocated to Groups 2 and 3 received LONGRANGE® (eprinomectin 5% w/v extended-release injection), once on Day 0 or on Day 109, respectively, at 1 ml/50 kg body weight (equivalent to 1 mg eprinomectin per kg). Treatments were administered by subcutaneous injection in the front of the shoulder on Day 0, when *Hypoderma* larvae were expected to be at the first larval stage (L1) or on Day 109, when warbles had been formed in at least six cattle of each of Groups 1 and 3, and *Hypoderma* larvae were expected to be at the second or third larval stage (L2/L3). For dose calculation, animals were weighed prior to treatment on the respective treatment days.

After allocation to treatment groups, cattle were held throughout the study indoors in two-loose pens on straw, fed roughage-based diet and were provided with water *ad libitum*.

Cattle were examined for warbles at intervals of approximately two weeks from Day 41 (January 23, 2017) until Day 69 and subsequently on a weekly basis until study end. Beginning on Day 118 and at each subsequent occasion, sufficiently mature *Hypoderma* larvae about to emerge were expressed from the warbles by gentle manual pressure and collected, and their viability was determined (motile larvae were considered alive). The cattle were examined until the last larval *Hypoderma* were collected after cutting open the remaining warbles (Day 148; May 10, 2017 = study end). The larvae were identified to larval instar and species on the basis of morphological characters [34, 35] unless identification was prevented by disintegration. All *Hypoderma* larvae were stored in 70% ethanol prior to and after identification.

### Data analysis

The primary effectiveness variable was the sum of live larval *Hypoderma* counts collected from Day 118 until all larvae were collected (Day 148). The sum of the counts was transformed to the natural logarithm count of (count + 1) for statistical analysis. For each LONGRANGE®-treated group, a separate analysis of variance model using the MIXED procedure in SAS v.9.4 was constructed, with the Treatment Group listed as a fixed effect and Pen listed as random effect. For each LONGRANGE®-treated group, the percent efficacy was calculated as 100[(C-T)/C], where C is the back-transformed least squares means from the statistical model in the saline-treated control group and T is the back-transformed least squares means from the statistical model in the LONGRANGE®-treated group.

## Data Availability

Data supporting the conclusions of this article are provided within the article. The raw data are available from the authors upon request.

## Abbreviations

L1: first-instar larva
L2: second-instar larva
L3: third-instar larva
